# Thermal performance augmentation in a pipe employing hybrid nanofluid and a plate as turbulator with V-shaped double-winglet ribs

**DOI:** 10.1038/s41598-024-57374-7

**Published:** 2024-03-28

**Authors:** Zhongmian Fan, Lingxiao Wang, Changjun Liu, Seyyed Amirreza Abdollahi

**Affiliations:** 1 College of Chemical Equipment, Shenyang University of Technology, Liaoyang, 111000 China; 2 Faculty of Mechanical Engineering, University of Tabriz, Tabriz, Iran

**Keywords:** Heat transfer enhancement, Passive method, Turbulator, Vortex generator, Nanofluid, Mechanical engineering, Fluid dynamics

## Abstract

This article employs a plate with V-shape ribs inside a tube as turbulator to augment the heat transfer rate. The utilized vortex generators are double-winglets arranged in a V-shape placed on both sides of the plate. The proposed system’s suggested working fluids are water-based hybrid nanofluids, including Al_2_O_3_–Cu/water, Cu–CuO/water, and Cu–TiO_2_/water. This work involves a numerical evaluation of the effects of the type and volume concentration of the examined hybrid nanofluids on the enhancement of heat transfer. The experimental results are used to validate the numerical model. It is worth mentioning that all the obtained numerical results are compared with the simple tube, without any turbulator (vortex generator) and in the presence of water instead of the hybrid nanofluids. Based on the numerical results, it can be concluded that all employed hybrid nanofluids showed improved thermal performance compared to pure water. Furthermore, the differences between the models are more substantial for higher Reynolds numbers than for lower Reynolds numbers. In Re = 30,000, the Cu–TiO_2_/water exhibits the lowest thermal performance improvement (augmentation of about 0.3%), while the Cu–CuO/water at Re = 50,000 exhibits the largest thermal performance improvement (augmentation of approximately 5.7%), in the case of ∅_1_ = ∅_2_ = 0.5%. For ∅_1_ = ∅_2_ = 1%, the Cu–TiO_2_/water at Re = 30,000 has the lowest thermal performance improvement (augmentation of around 1.1%), while the Cu–CuO/water at Re = 50,000 has the most thermal performance improvement (augmentation of roughly 8.7%). According to the augmentation of around 2.8% at Re = 30,000 for Cu–TiO_2_/water and approximately 10.8% at Re = 50,000 for Cu–CuO/water, the thermal performance increase in the scenario of ∅_1_ = ∅_2_ = 1.5% is the lowest. In Conclusion, the Cu–CuO/water hybrid nanofluid with a volume concentration of ∅_1_ = ∅_2_ = 1.5% has the greatest thermal performance value of all the hybrid nanofluids studied.

## Introduction

A heat exchanger is a device that transmits heat from one fluid to another, from a fluid to a solid surface, or from one solid surface to another. Heat exchangers are widely used in various applications, including power production, refrigeration, air conditioning, chemical processing, etc^1,2^.

The technique of enhancing the efficacy of heat exchangers is known as heat transfer augmentation. Depending on the kind and design of the heat exchanger, there are many approaches for improving heat transmission. Overall, there are two kinds of heat transfer augmentation methods: (1) passive and (2) active^3^.

Passive heat transfer augmentation techniques are approaches for increasing the heat transfer rate in a heat exchanger without requiring any extra power or energy. They adjust the heat exchanger's geometry, surface, or fluid characteristics to improve the heat transfer area, coefficient, or driving force. Passive techniques are often more dependable, cost-effective, and simple to adopt than active ones, which need the installation of extra devices or processes to improve heat transmission^4^.

Passive techniques are often more dependable, cost-effective, and simple to apply than active ones, which require additional devices or processes to improve heat transmission. Active techniques require external power or energy input, whereas passive ones do not^5^.

A literature review of recent works applying passive techniques to improve the heat transfer rate is presented here. In the work of Kazemi Moghadam et al.^6^, a solar air heater with novel arc-shaped ribs was taken into consideration. The suggested solar air heater's heat transfer method was mathematically assessed for Reynolds numbers ranging from 6000 to 12,000. The effects of the rib cross-section type, inter-rib distance, aspect ratio, and solar air heater's pitch were examined on the solar air heater's thermal efficiency. First, according to the results, ribs with quadrangular cross sections had the best performance coefficients. Additionally, ribs with an aspect ratio of 0.5 and a low pitch of 40 mm exhibit the best thermal performance. Additionally, rib spacing has little impact on the thermal effectiveness of the solar air heater under consideration.

Heat transmission and fluid flow in a corrugated coil tube with varying lobe-shaped cross-sections were quantitatively analysed by Zaboli et al.^7^. In addition, spiral twisted tape with various geometries is used as a turbulator in the suggested corrugate coil tube. The parameters investigated include the geometry of the cross-section of a corrugated coil and the geometry of twisted tape in a corrugated coil tube. The obtained data reveal that a five-lobe cross-section increases the Nusselt number and decreases pressure by 9.1% and 3.7%, respectively, compared to a three-lobe cross-section. Furthermore, increasing the area of spiral twisted tape in the five-lobe corrugated tube causes a 30.7% and 37.1% increase in Nusselt number and pressure drop, respectively.

Masoumpour-Samakoush et al.^8^ studied the melting process of phase change material (PCM) in storage by inserting creative fins that mix rectangular and triangular fins. The temperature of the left wall of storage is considered constant and more significant than the melting point of PCM. The effects of numerous efficiency factors on the melting process are investigated, including internal fin schematics, triangular fin height, hot wall temperature, and different types of PCM. The results indicated that the proposed one increases the melting rate by roughly 57.56% among the different analysed fin shapes. Melting time lowers as fins increase, resulting in a greater energy charging rate.

The heat transmission properties of humid air in a rectangular channel as a solar air heater fitted with V-shaped ribs were explored numerically by Nouri Kadijani et al.^9^. The effects of suggested rib geometrical parameters such as height, pitch, and angle relative to airflow direction, as well as relative humidity and Reynolds number of incoming humid air, on the thermal performance of the proposed solar air heater were examined. The numerical findings were obtained using the commercial CFD package, ANSYS FLUENT 18.2, which operates on a finite volume basis. The results reveal that using ribs causes the thermal barrier layer to detach and reconnect throughout various operating circumstances, eventually increasing the heat transfer rate.

Salhi et al.^10^ evaluated numerically the impact of employing two slightly inclined baffles of varying height and number on the heat transfer rate inside a horizontal channel. A forced convective flow of a cooling fluid (air) crosses the channel. This numerical study investigated the effects of baffle height and number on heat transfer rate enhancement. This system's mathematical model comprised nonlinear partial equations with exceedingly complicated analytical solutions, necessitating numerical analysis using a finite volume approach. The findings indicated that it is feasible to increase the thermal performance of the investigated system by using designs that allow for the highest heat transfer rate with the least amount of energy loss. Furthermore, at the lowest Reynolds number (Re = 10,000), the heat transfer rate increases by around 59.09% when the height of the baffles increases from 0.01 to 0.03 m (a 200% increase). Furthermore, raising the height of the baffles by up to 200% improves the heat transfer rate by roughly 50.53% at the maximum analysed Reynolds number (Re = 87,300). Furthermore, using more baffles increases heat transfer rates and a significant pressure decrease.

In Ajarostaghi et al.^11^ study, a novel turbulator was placed at the entry of the hydrogen side of a new form of heat exchanger known as a hydrogen preheater. The suggested turbulator was divided into two sections: (1) the conical component with lateral outputs and (2) the finned part with spiral blades. In the suggested preheater, the effects of four influential geometrical factors on hydrogen's hydrothermal behaviour were explored numerically. The number of blades, length, diameter, and angle of the blades were taken into consideration as geometrical characteristics. The obtained numerical results showed that using the suggested hydrogen preheater of the PEMFC improves the PEMFC's performance by lengthening the life of the membrane electrodes.

Afsharpanah et al.^12^ focused on employing various enhancement strategies, including using porous foams, fins, and nanomaterials, to accelerate one of these systems' phase change rate toward the desired speed. The impact of container orientation was initially researched for this purpose. The freezing of four distinct PCM-porous composites containing porous materials made of aluminium, copper, nickel, and graphite augmented with nanotechnology was then investigated. After testing several foam porosities, the copper foam was chosen as the best composite for the unit. It was discovered that although using CuO nanoparticles to fill 3%vol of the container's capacity only produces a 24.3% increase, using copper foam to serve the same volume speeds up the process by 84.6%.

Amoli et al.^13^ investigated the impact of the eccentricity of the porous medium on the findings. They assessed experimentally and mathematically the laminar forced convection flow inside a horizontal tube partly filled with a porous medium under constant heat flux. The governing equations were solved in three dimensions via a numerical analysis. Conformal mapping was used to transform the tube cross-section in the fluid domain (space between two eccentric circles) into a rectangle, simplifying the grid generation and satisfying the boundary conditions. The equations were then solved in a computational domain in this domain. The Darcy–Brinkman–Forchheimer model was used to simulate the hydrodynamic behaviour of the flow in the porous zone. The findings demonstrate that the eccentricity of the porous material affects both the pressure drop and the heat transfer coefficient concurrently. Naturally, the heat transfer coefficient reduction is less pronounced as the porous medium's thickness decreases. The average Nusselt number, for instance, falls by 66% for a reduced porous thickness at RP = 0.5 as the eccentricity of the porous medium grows up to E = 0.4. When Da = 10^5^ and E = 0.4, the pressure drop decrease is reduced by a maximum of 25%.

The impact of inserting a curved novel turbulator on improving heat transmission in a pipe was examined using a three-dimensional numerical assessment by Mousavi Ajarostaghi et al.^14^. The researched curved nozzle employs several rows of flow directors to produce turbulent swirl flows. The results show that the conical nozzle's output diameter has a negligible impact on thermal performance. Additionally, with a greater number of flow director rows, thermal performance and heat transmission are improved. Additionally, the average Nusselt number grows as the angle of curvature increases because the secondary flows are growing. Due to a significant pressure loss, the case with the suggested curved turbulator exhibits inferior thermal performance than the uncurved one. The case with d = 7 mm has the highest average Nusselt number.

Yousefi et al.^15^ presented a numerical analysis to examine the use of nanofluid in place of pure fluids in the microchannel heat sink with ribs and dimples as turbulator insertion within the backwards-facing step microchannel. Through a step microchannel that faces backwards, the lattice Boltzmann technique was used to examine the heat transfer and laminar flow behaviour of 4% concentration of Al_2_O_3_–water nanofluids. A steady heat flux was applied to the microchannel's bottom wall downstream of the step. On this area of the wall, there were ribs and hemispherical dimples that served as vortex generators. The findings indicated that, for Re = 40 and 100, respectively, an increase in the average Nusselt number from two to eight ribs (100% growth) results in proliferation of 63.64 and 64.65%. Additionally, an increase in rib height from 0.5H to 2H (300% growth) increased the average Nusselt number by roughly 54.54 and 40.91% at Re = 40 and 100, respectively. The impact of adding ribs on the Nusselt number can occasionally be negligible or undesired, especially when the numbers are smaller or the ribs are shorter.

Mousavi Ajarostaghi et al.^16^ investigated numerically a helical double-tube heat exchanger's hydrothermal equipped with a novel form of swirl generator, which had two sections: outward curving blades and a semi-conical portion with two holes in the inside part. For research, two geometrical elements were used: the length and the location of the swirl generator. The computations were done using ANSYS FLUENT 18.2, a commercial FVM package^17^. The numerical results suggest that a shorter swirl generator length improves hydrothermal behaviour. As a result, the model with L_1_ = 100 mm at m = 0.008 kg/s obtains the best thermal performance by about 17.65, 53.85, and 100% compared to the models with L_1_ = 200, 300 mm, and no swirl generator.

The effectiveness of using nanofluids as a passive method to improve heat transfer efficiency is due to their higher thermal conductivity, increased surface area for interaction with heated surfaces, disruption of boundary layers, uniform heat dispersion, fluid mixing promotion, and adaptability for different applications. These benefits increase heat transfer rates without the need for complicated systems, making nanofluids a flexible and scalable alternative for improving heat transfer in a variety of scenarios. In actual applications, stability and long-term performance issues must be considered^17–23^.

Ghazanfari et al.^24^ used numerical analysis to examine the effects of twisted tubes and Al_2_O_3_ nanofluid on the thermal performance of the shell and tube heat exchanger. They used two separate turbulence models (realizable *k*–*ε* and realizable *k*–*ε* 2nd order) in CFD. The current solution was validated by comparing the outcomes with the published study, which suggested the first-order realizable *k*–*ε* model as the appropriate approach. Additionally, pure water and the Al_2_O_3_ nanofluid at 5%,10%,15%, and 20% were used for twisted tubes and for smooth tube heat exchangers with six baffles, and for those without, only pure water was used. The findings showed that the pressure drop increased by roughly 14% when twisted tubes were used instead of smooth tubes, but the coefficient of heat transmission improved by nearly 20%. According to the data, using twisted tubes and 20% nanofluid simultaneously (as opposed to pure water and smooth tubes with six baffles) increases the heat transfer coefficient by roughly 8%. It decreases the pressure drop by almost 40%. In the other work, Ghazanfari et al.^25^ presented a three-dimensional CFD analysis to examine how nanofluids affect heat exchanger efficiency. Pitch lengths (P = 180, 135, 90, 67.5, and 45 mm) were analyzed to maximize the performance of twisted tubes. By comparing experimental and numerical data from earlier studies, the accuracy of the procedure was confirmed. The analysis covered a broad range of flow rates (0.5–2 kg/s), focusing on important variables such as heat transfer factors, outlet temperatures, and pressure decreases. Their results showed that although marginally raising pressure drop, employing nanofluids in twisted tubes greatly improves heat transmission. In particular, using 0.1 vol% Cu and 0.15 vol% Al_2_O_3_ nanoparticles in the twisted tube with a pitch length of 45 mm results in heat transfer enhancements of 1.04 and 1.12 times, respectively, compared to the smooth tube device with six baffles. Furthermore, removing baffles in favour of the ideal twisted tube arrangement yields a significant 1.55-fold decrease in pressure drop. These findings demonstrate the possibility of using nanofluids to improve heat exchanger performance and provide insightful information for designing and optimising heat transfer systems in various industrial settings.

This work uses a plate to improve the heat transfer inside the tube, in which vortex generators are placed in a V shape. The employed vortex generators are double-winglets in V-shaped arrays. The working fluid in the proposed system is water-based nanofluid. In this work, the impacts of the type and volume concentration of the considered nanofluids on the heat transfer enhancement in the tube are evaluated numerically. The employed numerical model was validated with the experimental results. Also, it is worth mentioning that all the obtained numerical results were compared with the simple tube, without any turbulator (vortex generator) and in the presence of water instead of nanofluid. A commercial CFD code has performed the numerical simulations, ANSYS FLUENT 19.2, which works based on the finite volume method (FVM).

## Problem description

**Figure 1:**
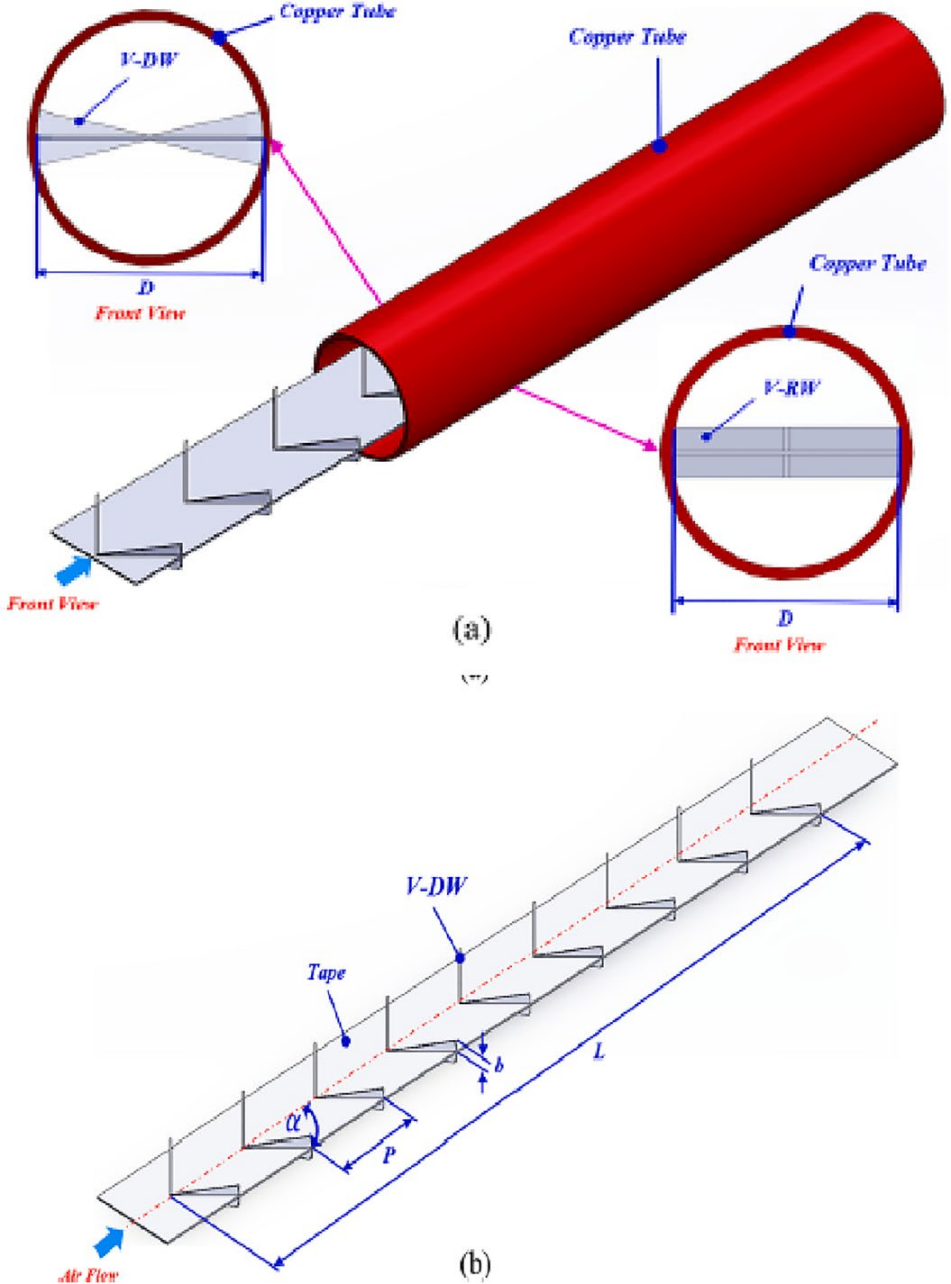
The schematics of the proposed tube equipped with the turbulator; (**a**) Tube, and (**b**) plate with double-winglet as vortex generator^26^.

The schematics of the proposed pipe equipped with a plate with some double-winglet as turbulators are demonstrated in Fig. 1 in various views. It is worth mentioning that the proposed plate as vortex generator is placed precisely at the centre of the tube, and the double-winglets are located on both sides of the plate, bottom and top sides. Moreover, it should be stated that employing this type of vortex generator was evaluated by Promvonge and Skullong^26^ experimentally and did not present the numerical analysis, which is very helpful here to understand the impact of the proposed turbulator on the fluid flow and streamline inside the tube. Furthermore, the present numerical model is validated using the experimental results of the reference work^26^ related to the same geometrical and operational conditions.

**Figure 2:**
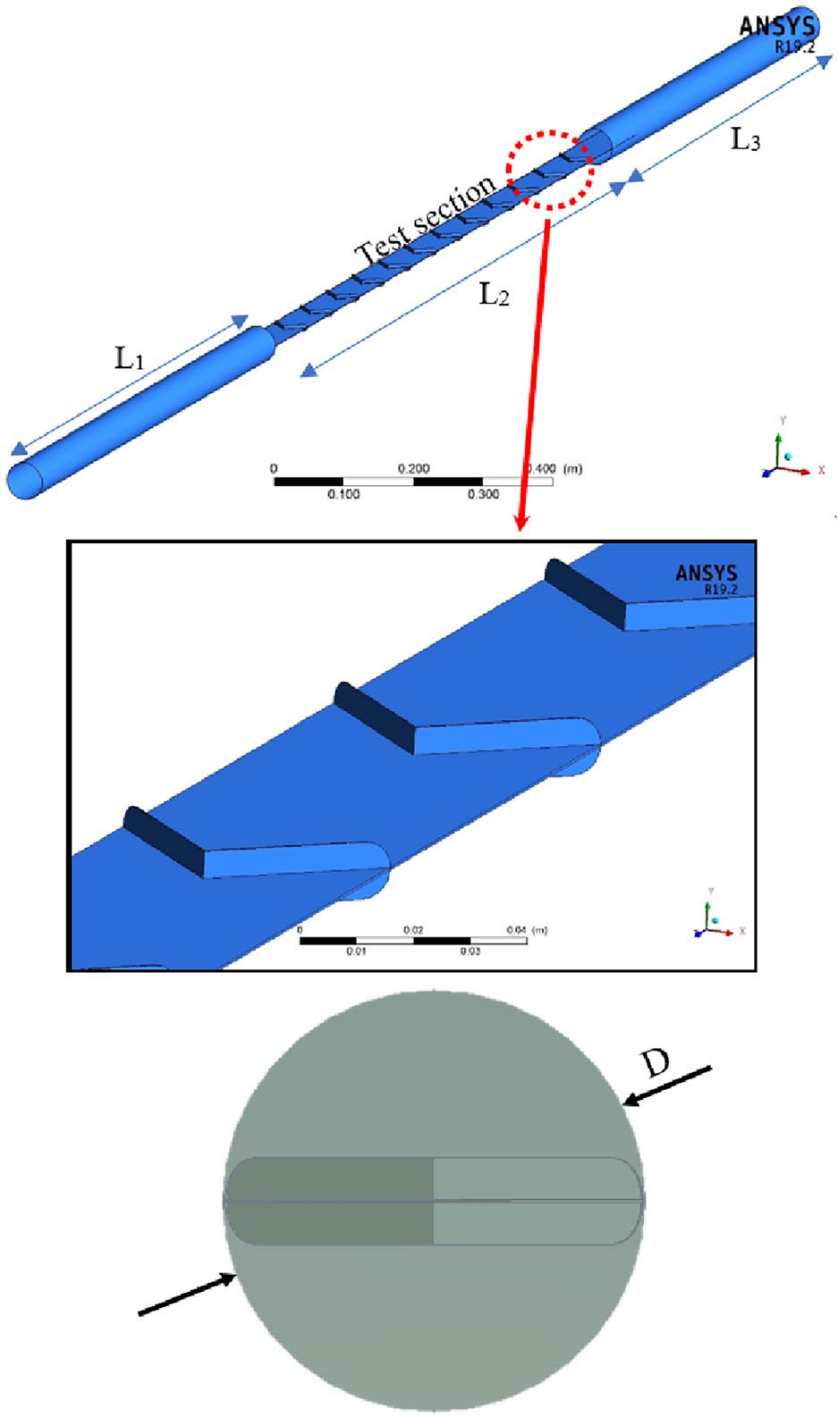
The schematics of the generated geometry of the proposed tube equipped with the turbulator for performing numerical simulations.

The schematics of generated geometry in various views for performing the numerical simulation are illustrated in Fig. 2. Accordingly, it can be seen that a clean channel (without turbulator) has been added at the entrance of the proposed heat exchanger to achieve the fully developed fluid flow (with a length of L_1_). A clean channel has been added at the end of the proposed heat exchanger for better numerical convergence (with a length of L_3_). The length of the tube with the proposed turbulator is L_2_. The dimensions of the geometrical factors are listed in Table 1.

**Table 1:** The dimensions of the geometrical factors.

Factors		Value
Diameter of the tube	D	50.8 mm
Length of the tube with turbulatort	L_2_	1200 mm
Length of the clean tube at inlet	L_1_	900 mm
Length of the clean tube at outlet	L_3_	900 mm
The pitch of the double-winglet	P	100 mm
The angle between the double winglet	α	45°

## Numerical modeling details

### Governing equations

Continuity:

1 $$\frac{\partial }{{\partial X_{i} }}\left( {\rho u_{i} } \right) = 0$$

Momentum:

2 $$\frac{\partial }{{\partial X_{j} }}\left( {\rho u_{i} u_{j} } \right) = - \frac{\partial P}{{\partial X_{i} }} + \frac{\partial }{{\partial X_{i} }}\left[ {\mu \left( {\frac{{\partial u_{i} }}{{\partial X_{j} }} + \frac{{\partial u_{j} }}{{\partial X_{i} }} - \frac{2}{3}\delta_{ij} \frac{{\partial u_{i} }}{{\partial X_{j} }}} \right)} \right] + \frac{\partial }{{\partial X_{j} }}\left( { - \rho \overline{{{\acute{u}}_{i} {\acute{u}}_{j} }} } \right)$$

Energy:

3 $$\frac{\partial }{{\partial X_{i} }}\left( {\left( {u_{i} \left( {E\rho + P} \right)} \right)} \right) = \frac{\partial }{{\partial X_{j} }}\left[ {\left( {\lambda + \frac{{C_{p} \mu_{t} }}{{Pr_{t} }}} \right)\frac{\partial T}{{\partial X_{j} }} + u_{i} \left( {\tau_{ij} } \right)_{eff} } \right] = 0$$

In Eq. (3), the expressions $(\tau_{ij} )_{eff}$ and E denote deviated stress tensor and total energy, respectively, and are distinct as follows:

4 $$E = C_{p} T - \left( {{P \mathord{\left/ {\vphantom {P \rho }} \right. \kern-0pt} \rho }} \right) + \left( {{{u^{2} } \mathord{\left/ {\vphantom {{u^{2} } 2}} \right. \kern-0pt} 2}} \right)$$

5 $$\left( {\tau_{ij} } \right)_{eff} = \left[ {\mu_{eff} \left( {\frac{{\partial u_{j} }}{{\partial X_{i} }} + \frac{{\partial u_{i} }}{{\partial X_{j} }}} \right) - \frac{2}{3}\mu_{eff} \frac{{\partial u_{i} }}{{\partial X_{j} }}\delta_{ij} } \right]$$

The following equations determined turbulent kinetic energy:

6 $$\frac{{\partial \left( {\rho u_{i} k} \right)}}{{\partial x_{i} }} = \frac{\partial }{{\partial x_{j} }}\left[ {\left( {\mu + \frac{{\mu_{t} }}{{\sigma_{k} }}} \right)\frac{\partial k}{{\partial x_{j} }}} \right] + G_{k} - Y_{k}$$

7 $$\frac{{\partial \left( {\rho u_{i} \omega } \right)}}{{\partial x_{i} }} = \frac{\partial }{{\partial x_{j} }}\left[ {\left( {\mu + \frac{{\mu_{t} }}{{\sigma_{\omega } }}} \right)\frac{\partial \omega }{{\partial x_{j} }}} \right] + G_{\omega } - Y_{\omega } + D_{\omega }$$

### Numerical procedure

Some assumptions have been considered for the proposed numerical model as follows:

- The steady-state condition is considered for solving the equations numerically.
- The fluid flow regime is turbulence, and the *k*–*ω SST* turbulence model is employed here.
- The material of the solid section (the turbulator and the tube`s thickness) is aluminium.
- The air as the fluid flow material is considered incompressible, and its thermophysical properties are listed in Table 2. It is worth mentioning that the final working fluids in this work are water-based hybrid nanofluids, and the air has been employed as working fluid just for validation analysis because, in the reference work^26^, the used working fluid was air.

**Table 2:** The thermophysical properties of air as the base working fluid and the nanoparticles.

Property		Value
Air		
Density (kg m^−3^)	ρ	1.225
Thermal conductivity (W (m K)^−1^)	k	0.0242
Specific heat (J (kg K)^−1^)	C_P_	1006.43
Viscosity (kg (m s)^−1^)	µ	1.785e^−5^
Cu		
Density (kg m^−3^)	ρ	8920
Thermal conductivity (W (m K)^−1^)	k	398
Specific heat (J (kg K)^−1^)	C_P_	385
Viscosity (kg (m s)^−1^)	µ	–
CuO		
Density (kg m^−3^)	ρ	6350
Thermal conductivity (W (m K)^−1^)	k	69
Specific heat (J (kg K)^−1^)	C_P_	535
Viscosity (kg (m s)^−1^)	µ	–
Al_2_O_3_		
Density (kg m^−3^)	ρ	2710
Thermal conductivity (W (m K)^−1^)	k	205
Specific heat (J (kg K)^−1^)	C_P_	900
Viscosity (kg (m s)^−1^)	µ	–
TiO_2_		
Density (kg m^−3^)	ρ	4175
Thermal conductivity (W (m K)^−1^)	k	8.4
Specific heat (J (kg K)^−1^)	C_P_	692
Viscosity (kg (m s)^−1^)	µ	–

The details of employed numerical schemes are presented in Table 3.

**Table 3:** Employed numerical schemes in the present work.

Spatial discretization	
Gradient	SIMPLE pressure–velocity coupling
Pressure	Second order
Momentum	Second order upwind
Energy	Second order upwind
Residual	
Continuity	0.001
X, Y, and Z velocity	0.001
Energy	1.e^−6^

### Boundary conditions

There are three main boundary conditions. The wall of the tube (the section including turbulator) has a constant heat flux of 2000 W m^−2^. The boundary condition for the tube's inlet is *Velocity_Inlet,* and four inlet Reynolds numbers have been considered. Also, the inlet temperature of 300 K has been considered for the tube's inlet. The boundary condition for the outlet of the tube is *Pressure_Outlet*.

### Grid independence examination

**Figure 3:**
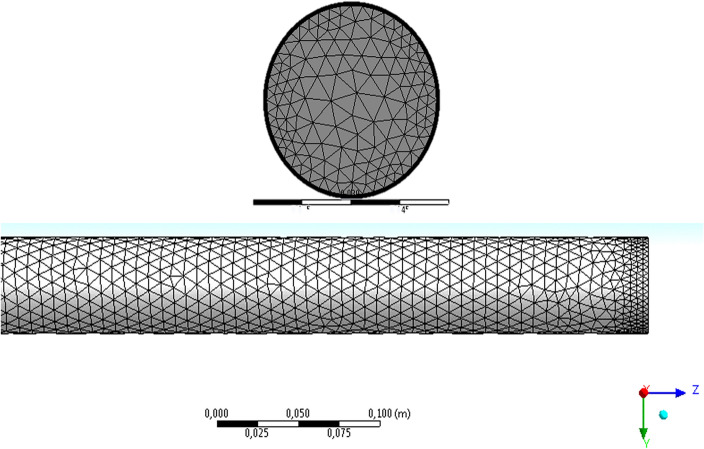
The pictures of the generated grid.

The pictures of the generated grid are demonstrated in Fig. 3. It should be noted that the boundary layer grid was considered for the outer wall of the tube. The number of cells in the generated grid is about 500,000 cells.

**Figure 4:**
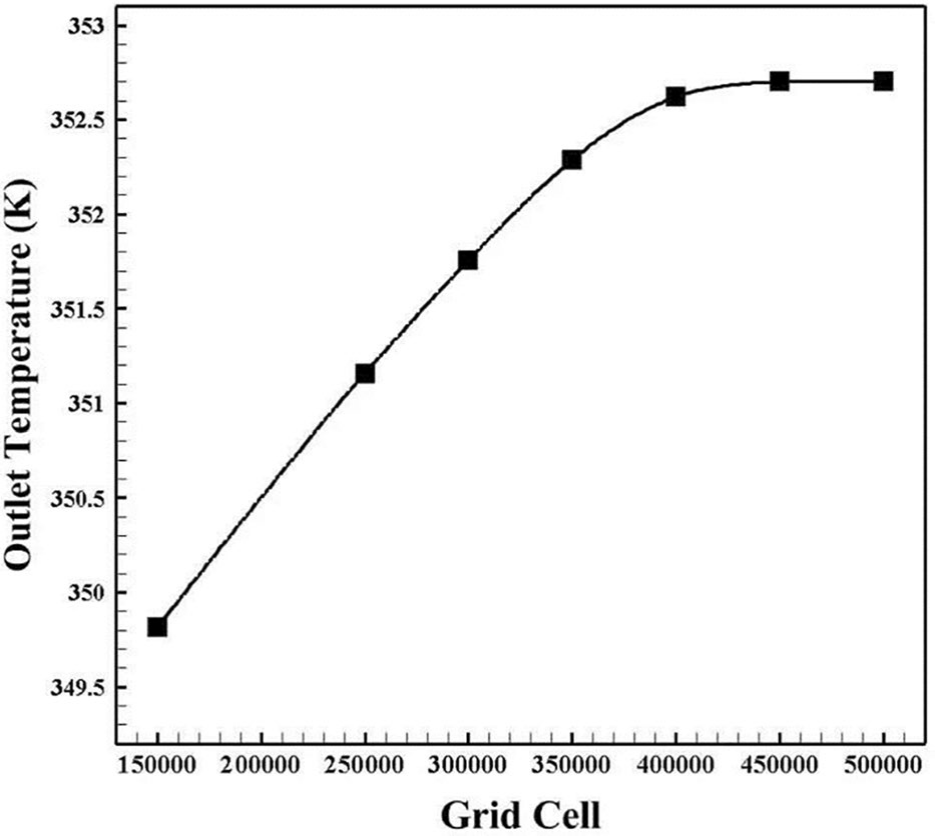
The outcomes of the grid independence analysis.

Seven scenarios with various cell numbers were considered for the grid independence analysis. The control parameter is the outlet temperature. Figure 4 presents the findings from the mesh independence analysis. Consequently, it is evident that there are so minor changes between grids that have roughly 400,000, 450,000, and 500,000 cells. Therefore, a grid of 400,000 cells was chosen for the simulations to reduce the time and expense involved.

## Results and discussions

### Validation analysis

**Figure 5:**
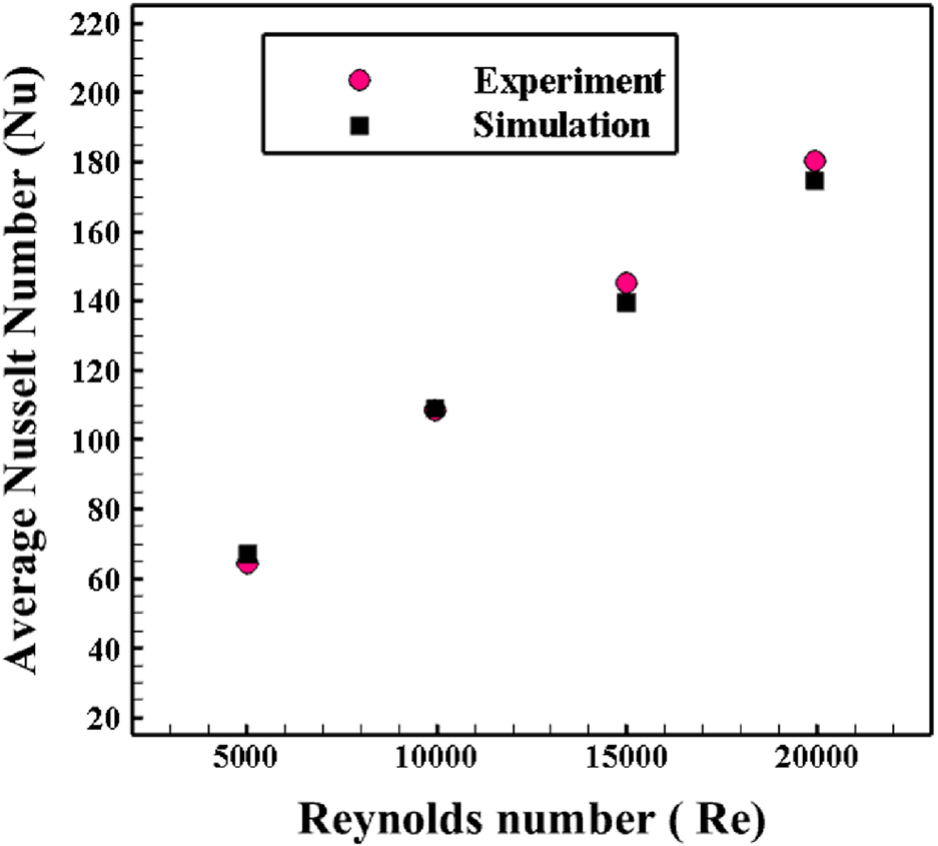
The results of the validation analysis of the employed numerical model with the experimental results of Promvonge and Skullong^26^.

In the validation study, the closest geometric and operational conditions to the proposed problem should be considered to prove the correctness of the employed numerical model. Accordingly, to verify the accuracy of the utilized model, the experimental results of Promvonge and Skullong^26^ have been considered. The obtained numerical results of the present work are compared with experimental results in Fig. 5. The profile of the average Nusselt number versus the Reynolds number has been considered as a control factor. Four Reynolds numbers have been considered here, including Re = 5000, 10,000, 15,000, and 20,000. Accordingly, it can be concluded that the employed numerical model is accurate.

**Figure 6:**
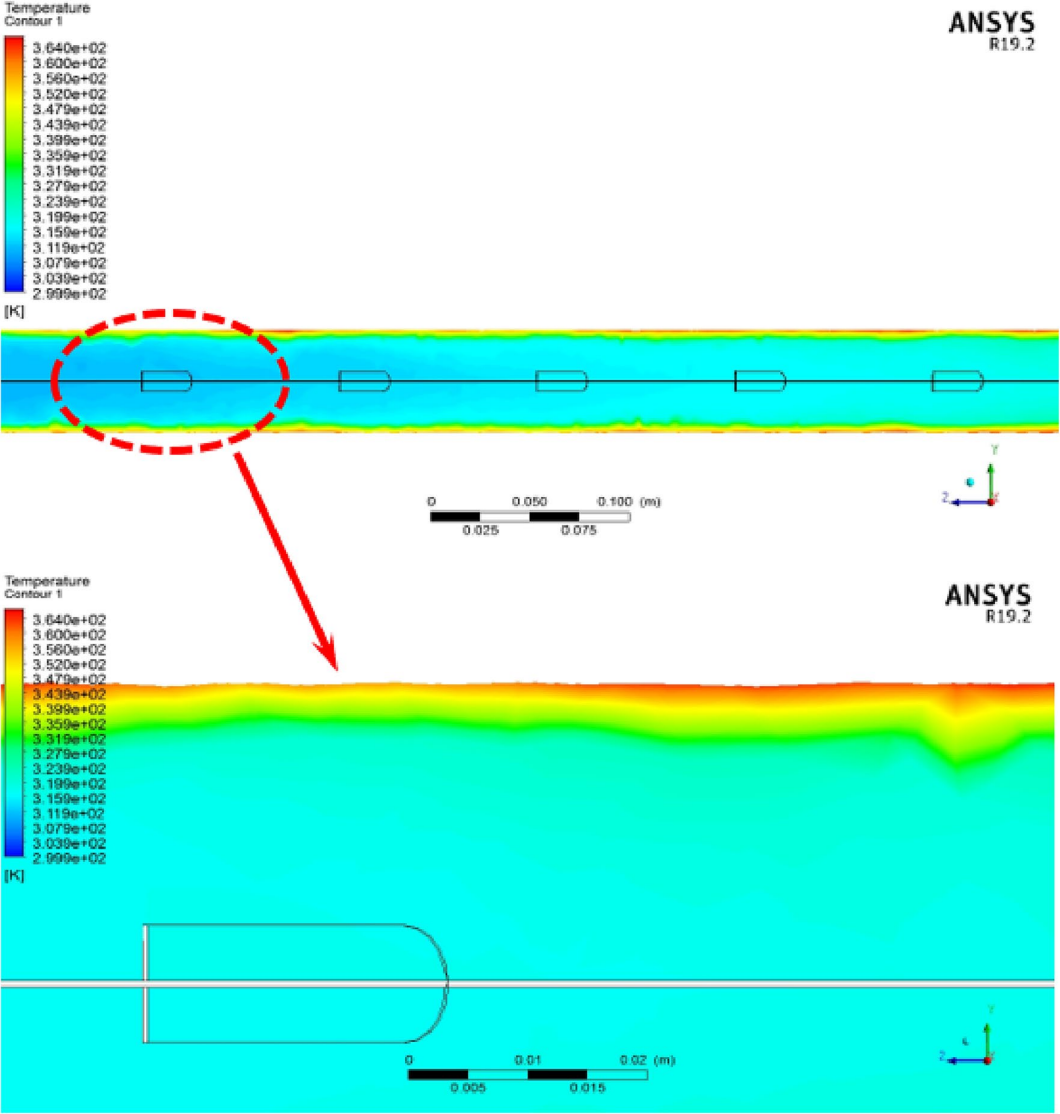
The temperature contour at the surface of X = 0 m inside the computational domain.

To understand better the impact of employing the proposed turbulator, double-winglet with a V-shaped arrangement, the temperature contour at the surface of X = 0 m inside the computational domain is illustrated in Fig. 6. As can be seen, after passing through the turbulator, the fluid becomes more turbulent, hits the pipe wall, and heats up, and vice versa. As can be seen, the fluid enters the tube at a low temperature (the blue part in the upper contour). After passing through the proposed V-shaped turbulator with a double winglet, its temperature increases and exits from the end of the tube with a higher temperature.

**Figure 7:**
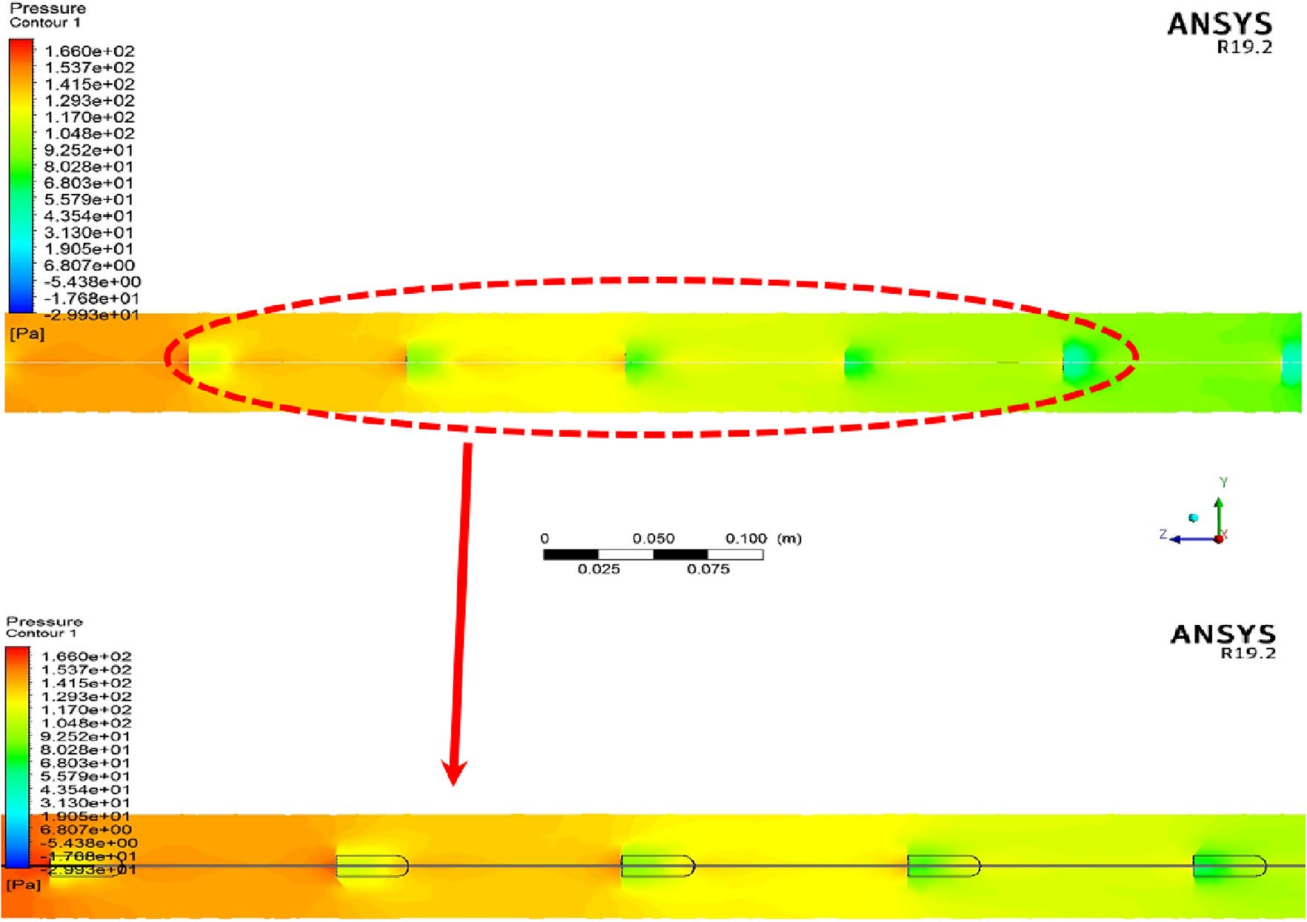
The pressure contour at the surface of X = 0 m inside the computational domain.

Using passive methods as an effective method to improve the heat transfer rate in different thermal systems, although it improves the thermal characteristics of the system in question, such as the amount of heat transferred and also temperature distribution, on the other hand, because these methods cause the formation of secondary and rotating flows (vortex), the result of which is a significant pressure drop in the considered system, and consequently, the cost of pumping the fluid also rises. For this purpose, when checking and analyzing the impact of using these methods in a thermal system, the pressure drop should also be checked simultaneously with the thermal parameters. The pressure contour at the surface of X = 0 m inside the computational domain is illustrated in Fig. 7. As can be seen, after the passing of fluid flow from the turbulator, the velocity of cold air flow decreases in front of the turbulators; at this point, the pressure has the highest value (red colour), and at the back sides of the turbulators, the value of pressure decreases that this green colour becomes noticeable. In other words, although using a turbulator leads to the creation of swirling flows, which is the most important factor in improving heat transfer in thermal systems, it creates a significant pressure drop, which is clearly seen in Fig. 7. It can be seen that the turbulators act like a barrier, and the fluid experiences a severe pressure drop after hitting them.

**Figure 8:**
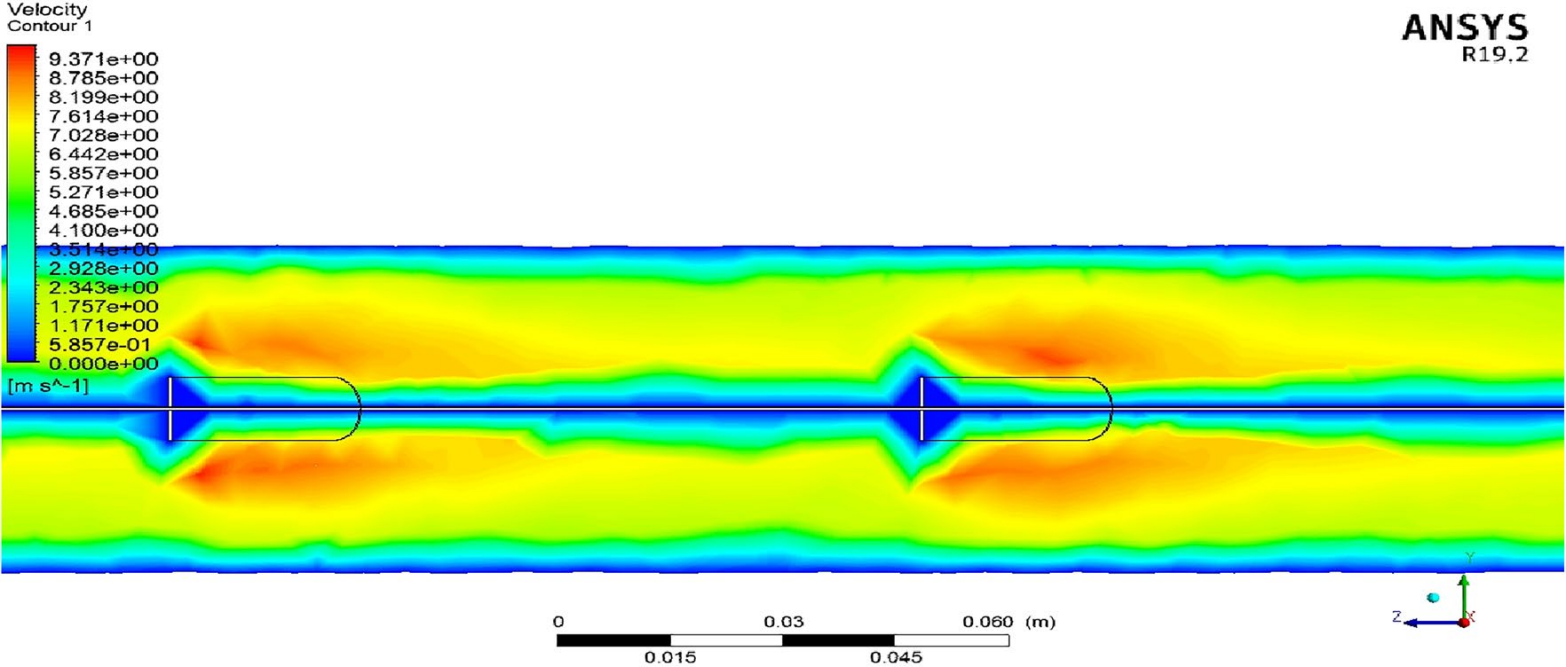
The pressure contour at the surface of X = 0 m inside the computational domain.

To realize better the influence of employing the proposed turbulator on the velocity distribution and creating the vortex flows, the velocity magnitude contour at the surface of X = 0 m inside the computational domain is illustrated in Fig. 8. As observed in this contour, the air's velocity decreases after encountering the intended turbulator and increases after passing through it. As evident, the highest velocity occurs behind the turbulator, where vortices are formed and are prominently visible. The fluid experiences a significant pressure drop upon impacting the turbulators (as shown in Fig. 7). Alternatively, because of the unique geometry of the turbulators, the fluid becomes trapped behind and in front of the turbulator, leading to the creation of rotating flows (vortex) (as shown in Fig. 8) that increases the heat transfer between the fluid and the hot wall in those regions.

### Impact of the hybrid nanofluid type

This section investigates the effect of the hybrid nanofluid type on heat transfer in the tube with the proposed turbulator under constant heat flux conditions. Three types of hybrid nanofluids, including Al_2_O_3_–Cu, Cu–CuO, and Cu–TiO_2_, have been examined, and the numerical results have been compared with those obtained from pure water. The results have been presented for four Reynolds numbers: 30,000, 35,000, 40,000, and 50,000. Additionally, numerical simulations for each type of hybrid nanofluid have been performed at three different volume concentration levels of the nanofluid: ∅_1_ = ∅_2_ = 0.5%, ∅_1_ = ∅_2_ = 1%, and ∅_1_ = ∅_2_ = 1.5%.

**Figure 9:**
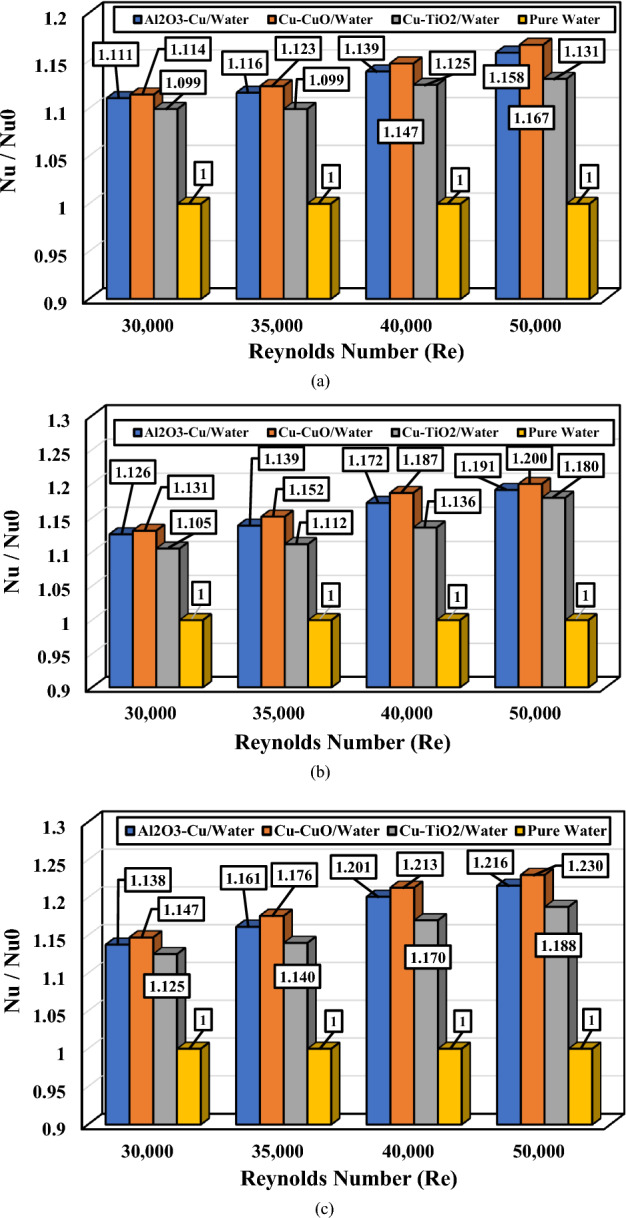
The value of the average Nusselt number ratio (Nu/Nu_0_) as a function of Reynolds number for different hybrid nanofluids; (**a**) ∅_1_ = ∅_2_ = 0.5%, (**b**) ∅_1_ = ∅_2_ = 1.0%, and (**c**) ∅_1_ = ∅_2_ = 1.5%.

The average Nusselt number ratio (Nu/Nu0) for different Reynolds numbers, nanofluids, and various volume concentrations are depicted in Figs. 9. It can be observed that even though the differences in results are marginal, all examined nanofluids exhibit higher Nusselt numbers compared to pure water. Cu–CuO demonstrates the highest Nusselt number among the studied nanofluids, while Al_2_O_3_–Cu ranks second. Notably, the disparity between models is more pronounced in this case than in other scenarios.

**Figure 10:**
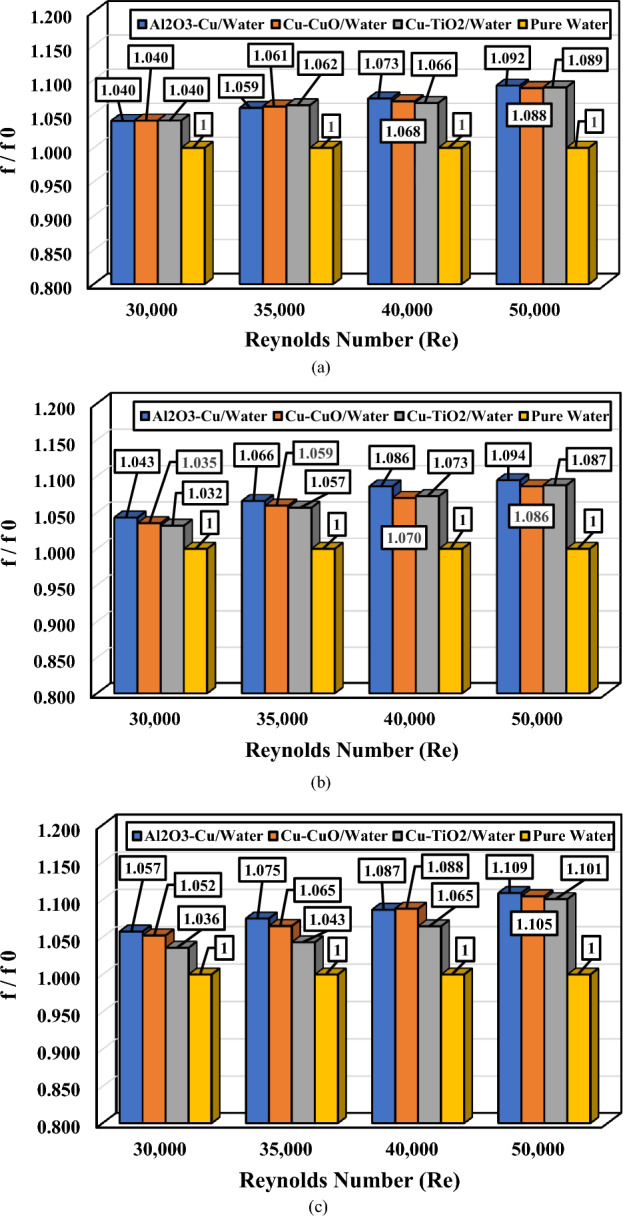
The ratio of f/f_0_ for different Reynolds numbers and various nanofluids; (**a**) ∅_1_ = ∅_2_ = 0.5%, (**b**) ∅_1_ = ∅_2_ = 1.0%, and (**c**) ∅_1_ = ∅_2_ = 1.5%.

The ratio of friction factor (f/f_0_) for different Reynolds numbers, nanofluids, and varying volume concentrations is illustrated in Fig. 10. As observed in Fig. 10, for all cases, the friction factor ratio (f/f_0_) is more significant than one for all examined nanofluids. This implies that the utilization of nanofluids leads to an increase in the friction factor compared to pure water. As evident, the nanofluid Al_2_O_3_–Cu exhibits the highest friction factor among the working fluids.

**Figure 11:**
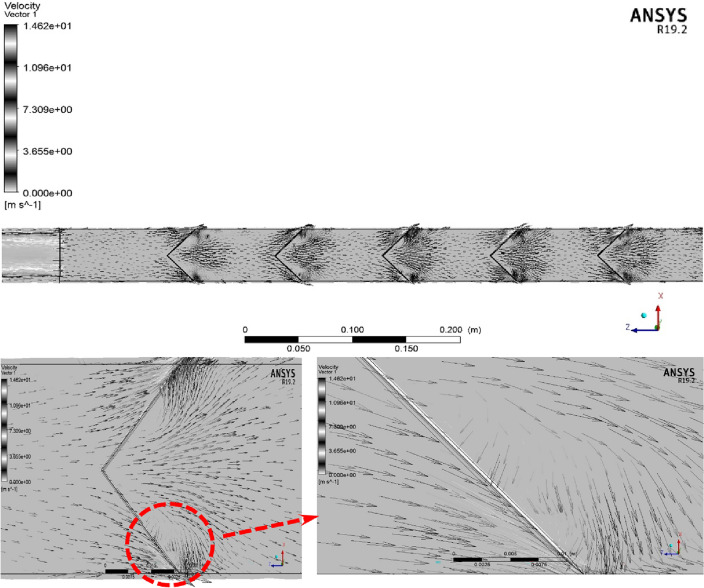
The vortices generated behind the turbulators.

The vector of fluid flow on the inserted plat and its winglets inside the tube as turbulators is demonstrated in Fig. 11. Accordingly, it can be seen that the presence of the proposed winglets results in the creation of vortex and secondary flows behind them and consequently, the heat transfer rate increases.

**Figure 12:**
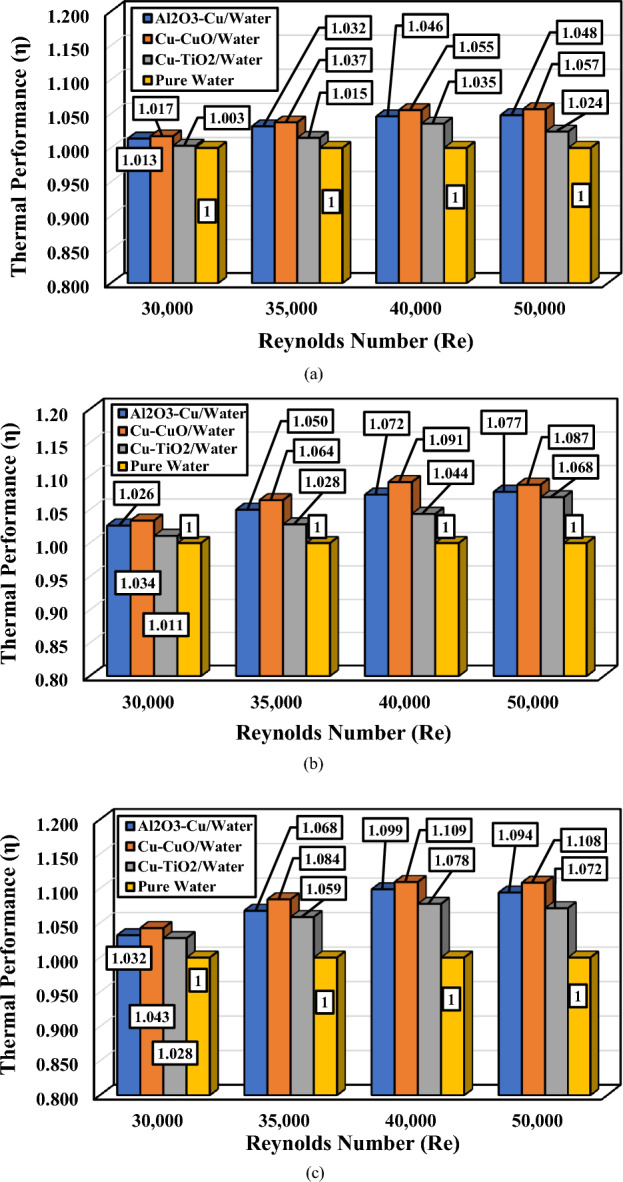
The variation of thermal performance versus the Reynolds number for various hybrid nanofluids; (**a**) ∅_1_ = ∅_2_ = 0.5%, (**b**) ∅_1_ = ∅_2_ = 1.0%, and (**c**) ∅_1_ = ∅_2_ = 1.5%.

The best parameter for considering both the heat transfer parameter and pressure drop factor is thermal performance, which is calculated by η = (Nu/Nu_0_)/(f/f_0_)^1/3^. In this correlation, the index 0 refers to the model with pure water. The higher value of thermal performance results in a higher ratio of the average Nusselt number and a lower value of the friction factor ratio (or pressure drop). The variation of thermal performance versus the Reynolds number for various hybrid nanofluids and volume concentration of nanoparticles is illustrated in Fig. 12.

Figure 12 illustrates that all the employed hybrid nanofluids have higher thermal performance than pure water. Moreover, it can be seen that the differences between the models in higher Reynolds numbers are more significant than the lower Reynolds numbers. Among all the considered hybrid nanofluids, the most significant thermal performance value belongs to the Cu–CuO at the volume concentration of ∅_1_ = ∅_2_ = 1.5%.

For instance, in the case of ∅_1_ = ∅_2_ = 0.5%, at the lowest considered Reynolds number (Re = 30,000), the thermal performance of the hybrid nanofluids of Al_2_O_3_–Cu, Cu–CuO, and Cu–TiO_2_ is 1.3, 1.7, and 0.3% more than the pure water, respectively. At the highest considered Reynolds number (Re = 50,000), the thermal performance of the hybrid nanofluids of Al_2_O_3_–Cu, Cu–CuO, and Cu–TiO_2_ is 4.8, 5.7, and 2.4% more than the pure water, respectively.

Also, in the case of ∅_1_ = ∅_2_ = 1%, at the lowest considered Reynolds number (Re = 30,000), the thermal performance of the hybrid nanofluids of Al_2_O_3_–Cu, Cu–CuO, and Cu–TiO_2_ is 2.6, 3.4, and 1.1% more than the pure water, respectively. At the highest considered Reynolds number (Re = 50,000), the thermal performance of the hybrid nanofluids of Al_2_O_3_–Cu, Cu–CuO, and Cu–TiO_2_ is 7.7, 8.7, and 6.8% more than the pure water, respectively.

Moreover, in the case of ∅_1_ = ∅_2_ = 1.5%, at the lowest considered Reynolds number (Re = 30,000), the thermal performance of the hybrid nanofluids of Al_2_O_3_–Cu, Cu–CuO, and Cu–TiO_2_ is 3.2, 4.3, and 2.8% more than the pure water, respectively. At the highest considered Reynolds number (Re = 50,000), the thermal performance of the hybrid nanofluids of Al_2_O_3_–Cu, Cu–CuO, and Cu–TiO_2_ is 9.4, 10.8, and 7.2% more than the pure water, respectively.

## Conclusion

This study used a plate to increase heat transmission within a tube, using vortex generators arranged in a V form. The vortex generators used are double-winglets arranged in V-shaped arrays. The suggested system's working fluid is a water-based nanofluid. The effects of the type and volume concentration of the nanofluids under consideration on heat transfer enhancement in the tube are quantitatively examined in this work. The experimental data were used to validate the numerical model that was used. It is also worth noting that the numerical findings obtained were compared with a basic tube, without any turbulator (vortex generator), and with water instead of nanofluid. The numerical findings are as follows:

- Hybrid nanofluids have better thermal performance than pure water.
- Furthermore, the discrepancies between the models at higher Reynolds numbers are more substantial than at lower Reynolds numbers.
- In the case of ∅_1_ = ∅_2_ = 0.5%, the lowest thermal performance improvement belongs to the Cu–TiO_2_ at Re = 30,000 by augmentation of about 0.3%, and the highest thermal performance improvement belongs to the Cu–CuO at Re = 50,000 by augmentation of about 5.7%.
- In the case of ∅_1_ = ∅_2_ = 1%, the lowest thermal performance improvement belongs to the Cu–TiO_2_ at Re = 30,000 by augmentation of about 1.1%, and the highest thermal performance improvement belongs to the Cu–CuO at Re = 50,000 by augmentation of about 8.7%.
- In the case of ∅_1_ = ∅_2_ = 1.5%, the lowest thermal performance improvement belongs to the Cu–TiO_2_ at Re = 30,000 by augmentation of about 2.8%, and the highest thermal performance improvement belongs to the Cu–CuO at Re = 50,000 by augmentation of about 10.8%.
- The Cu–CuO/water hybrid nanofluid with a volume concentration of ∅_1_ = ∅_2_ = 1.5% has the greatest thermal performance value of all the hybrid nanofluids studied.

## Data Availability

The datasets used and/or analyzed during the current study available from the corresponding author on reasonable request.
